# Variability of Single Pulse Electrical Stimulation Responses Recorded with Intracranial Electroencephalography in Epileptic Patients

**DOI:** 10.1007/s10548-022-00928-7

**Published:** 2022-12-15

**Authors:** Maciej Jedynak, Anthony Boyer, Blandine Chanteloup-Forêt, Manik Bhattacharjee, Carole Saubat, François Tadel, Philippe Kahane, Olivier David, F-TRACT Consortium

**Affiliations:** 1 Grenoble Institut Neurosciences, Université Grenoble Alpes, Inserm, U1216, 38000 Grenoble, France; 2 Aix Marseille Université, Inserm, INS, Institut de Neurosciences des Systèmes, Marseille, France; 3 Neurology Department, CHU Grenoble Alpes, Grenoble, France; 4 Signal and Image Processing Institute, University of Southern California, Los Angeles, USA

**Keywords:** Brain atlas, Connectivity mapping, Stereoelectroencephalography (SEEG), Cortico-cortical evoked potentials (CCEP), Single pulse electrical stimulation (SPES)

## Abstract

Cohort studies of brain stimulations performed with stereo-electroencephalographic (SEEG) electrodes in epileptic patients allow to derive large scale functional connectivity. It is known, however, that brain responses to electrical or magnetic stimulation techniques are not always reproducible. Here, we study variability of responses to single pulse SEEG electrical stimulation. We introduce a second-order probability analysis, i.e. we extend estimation of connection probabilities, defined as the proportion of responses trespassing a statistical threshold (determined in terms of Z-score with respect to spontaneous neuronal activity before stimulation) over all responses and derived from a number of individual measurements, to an analysis of pairs of measurements.

Data from 445 patients were processed. We found that variability between two equivalent measurements is substantial in particular conditions. For long ( > ~ 90 mm) distances between stimulating and recording sites, and threshold value Z = 3, correlation between measurements drops almost to zero. In general, it remains below 0.5 when the threshold is smaller than Z = 4 or the stimulating current intensity is 1 mA. It grows with an increase of either of these factors. Variability is independent of interictal spiking rates in the stimulating and recording sites.

We conclude that responses to SEEG stimulation in the human brain are variable, i.e. in a subject at rest, two stimulation trains performed at the same electrode contacts and with the same protocol can give discrepant results. Our findings highlight an advantage of probabilistic interpretation of such results even in the context of a single individual.

## Introduction

Single pulse electrical stimulation (SPES) of the human brain allows identification of epileptogenicity and connectivity of cortical areas (Lacruz et al., 2007; David et al. 2010). It is performed in pharmaco-resistant focal epilepsy patients implanted with depth electrodes in preparation for brain surgery (Valentin, 2002; Cuello Oderiz et al., 2019). Neuronal responses to stimulation (SPES-R) are recorded by subdural electrocorticographic or stereo-electroencephalographic (SEEG) electrodes. These responses convey, inter alia, information about the strength of directed functional connections from the stimulation to the recording sites as well as about the latency of signals propagation. In recent years we integrated SEEG stimulation data of over a thousand patients in the Functional Tractography (F-TRACT) database (https://f-tract.eu).

Group analysis performed on this database allows derivation of brain maps describing various features of connectivity in a given parcellation. For example, probability of connection between two parcels is defined as the proportion of significant responses recorded in the destination parcel over the number of all recordings in this parcel performed during stimulation applied in the source parcel. A significant response is such that it crosses within a given time window a statistical threshold, derived from spontaneous pre-stimulus neuronal activity. Similarly, a latency map contains median values of signal propagation times between the parcels. We have recently derived maps for a number of such features and shared them online on the F-TRACT website as a whole-brain connectivity atlas (Lemaréchal et al., 2022; Trebaul et al., 2018). This atlas is complemented with dependencies of the features (e.g. probability or latency) on factors such as the statistical threshold value, distance between stimulating and recording sites or stimulating current intensity (Trebaul et al., 2018).

The results published in such a probabilistic atlas are of the first-order, i.e. they provide an aggregate feature value, optionally with a measure of its dispersion, but do not inform about variability or repeatability of individual measurements. At the same time, it is known from animal and human studies that brain responses to electrical and magnetic single pulse stimulations, or brain responsiveness, depend on the brain state, the marker of which can be a consciousness level (e.g. as modulated by either sleep, anesthesia or attention) (Casali et al. 2013; Pigorini et al., 2015; Arena et al. 2021) or some pathological process, e.g. epileptic seizure (David et al., 2008; Saillet et al., 2013). Therefore, in the context of SEEG studies, a question about *intrinsic* variability of probabilistic connections arises: if an observed response to stimulation is above (below) a statistical threshold, would it remain (in)significant if evoked again after some period of time? A hypothesis that the significance of this response can change, is supported by relatively high false positive and false negative rates reported in some SEEG studies (Keller et al. 2011). The above question impacts interpretation of results published in a connectivity atlas. Say the probability of a connection is 0.5: does it mean that in half of the subjects this link always manifests as significant and in the other half always as insignificant (no intrinsic variability)? Or rather in all subjects it is significant in half of the performed measurements (large intrinsic variability)? Furthermore, what factors – if any – does intrinsic variability depend on? If these factors can be controlled, then in future studies variability could be, to some extent, modulated.

In this paper, we address these questions by performing a second-order analysis that considers variability between two identical measurements which will be referred to as measurement pairs. We analyze a series of such pairs, for each of them assessing consistency of significance of the two paired SPES-R. Importantly, both measurements in a pair were performed in the same experimental conditions. Stimulation parameters, data recording and analysis procedures are invariant between the two measurements, therefore variability between the corresponding responses originates in the brain activity itself. It is coherent with the fact that brain states during which signals were recorded are not guaranteed to be identical, though the patients were awake, at rest and asked to lay still. In this study, we do not explore the ample variability of response waveforms, but instead we focus only on the variability of their significance. Thereby we adhere to the methodology employed in the derivation of the F-TRACT atlas which detects the presence of a significant N1 component (defined as the first component arising before 200 ms post-stimulation) in the cortico-cortical evoked potentials after Z-scoring with pre-stimulation baseline (Lemaréchal et al., 2022; Trebaul et al., 2018).

Furthermore, we study how intrinsic variability of the presence of the N1 can be modulated by a choice of experimental parameters. We test for it by quantifying its dependence on the experimental setup, in particular how it changes with: (1) the chosen value of the statistical threshold (Z-score), (2) stimulating current intensity and (3) distance between the stimulating and recording electrode contacts. The choice of these factors links to our earlier work (Trebaul et al., 2018) and extends its results to the second order. Since all SEEG signals used for this work were recorded in epileptic patients, we complement this study with a verification whether our results depend on occurrence of interictal epileptic spikes in recorded signals.

## Materials and Methods

This study was performed on the F-TRACT database (https://f-tract.eu/), that to this day contains aggregated data of over one thousand patients with drug-resistant epilepsy who underwent SEEG implantations in preparation for resective brain surgery. Individual recordings, due to data privacy protection, are not shared. The code can be shared upon request. All patients gave consent to undergo invasive recordings and stimulation, and to share their data to the F-TRACT project operating under ethical guidelines of the International Review Board at INSERM (protocol number: INSERM IRB 14–140) for conducting international multicenter post-processing of clinical data. The procedure involved 1 Hz electrical stimulations of awake patients and simultaneous recording of responses in all electrodes (David et al., 2013; Trebaul et al., 2018). From the whole dataset, we selected those stimulations that were performed twice in the same technical conditions, i.e. delivered with the same electrode contacts, with the same stimulating frequency, current intensity, pulse pattern (monophasic vs. biphasic) and pulse duration, and within the same sessions (i.e. at a few minutes interval). There were 7079 such stimulation pairs (a stimulation pair consists of two identical stimulations) in 459 implantations (229 males, age 25 ± sd 14 and 227 females age 25.5 ± sd 13, three unspecified) of 445 patients from 22 medical centers. If a stimulation was performed three times, we considered two pairs. A *stimulation* consists of a train of pulses; we did not require the number of these pulses to be identical in paired stimulations. During each stimulation, signals from a number of electrode contacts are recorded, therefore one stimulation corresponds to many measurements. Two measurements performed by the same (bipolar) contact during two repeated stimulations constitute a measurement pair. We analyzed 566,371 such measurement pairs. We emphasize that the term “pair” does not refer to the fact that a stimulation is usually performed between two adjacent contacts, nor to the fact that the recording is later re-referenced to bipolar montage, also involving two adjacent contacts.

In order to provide comparability of our research with typical SPES-R studies, we apply here a fairly standard signal processing pipeline described in detail in Trebaul et al., 2018. In short, first signals were re-referenced to bipolar montage, since it is the most common choice in both clinical (Lachaux et al., 2003) and scientific (Zaveri et al., 2006) applications. Then, stimulation artifacts were removed (Trebaul et al., 2016) and signals were band-pass filtered between 1 and 45 Hz. Responses to all pulses in one stimulation run were averaged. The 800 ms post-stimulation averaged signal was then Z-scored with respect to the baseline defined as activity on the pre-stimulation [-400, -10 ms] interval. A SPES-R is considered significant if within the 800 ms it goes above a Z-score threshold, for which we used values from Z = 3 to Z = 7. We present the results for this wide time window and additionally, in order to provide translatability of our results into the context of above-mentioned probabilistic atlas, we performed the same analysis with a condition used in the atlas generation procedure. This condition is meant to reduce spurious signals and the number of indirect (polysynaptic) interactions, and it states that a response is considered significant only if the maximum of the peak crossing the threshold appears within a 200 ms time window after stimulation.

Assessment of the degree to which epileptic-like activity could manifest in recorded signals was based on the rate of interictal spikes appearing in time courses, as detected by the DELPHOS (Detector of Electro Physiological Oscillations and Spikes) software (Roehri et al., 2016, 2017). We verify if our results are contingent on the spiking rate as observed in the stimulating or recording contacts. Stimulating contacts are not recorded during stimulation, but the rates could be still derived from other sessions where these contacts served as the recording ones. Parameters set in DELPHOS are: power threshold (200), time ratio threshold (1.5) and frequency spread threshold (9). Because spike rate distributions vary for different individuals, we standardized them to Z-score by subtracting the mean and dividing by the standard deviation as obtained for each patient independently.

In the context of a single measurement pair, one of the three outcomes can be observed: (1) both readouts are above the statistical threshold ( “aa”), (2) both readouts are below the threshold (“bb”), (3) one readout is above and the other below the threshold (“ab”). In the context of many measurement pairs, we computed three normalized frequentist probabilities p_aa_, p_bb_, p_ab_ that correspond respectively to proportions of occurrences of each of the three above mentioned outcomes among all measurement pairs. p_ab_ quantifies intrinsic variability and takes values from zero to maximal intrinsic variability p_ab_^max^ that corresponds to independence and hence lack of correlation between measurements in a pair. We verified that probabilities of significance (computed over all pairs) of the first and the second paired measurement are equal, thus described with the same first-order probability p_a_ (such as the one published in the atlas). This equality together with independence of measurements leads to p_ab_^max^ = 2 · p_a_ · (1 - p_a_), where p_a_ = p_aa_ + 0.5 · p_ab_.

The sizes of groups we compare here do not have to be equal. For example, there are more measurements performed with some given current intensity value than with another value. In order to verify if this sampling bias does not affect the presented results, as well as in order to estimate their uncertainty, we employed a subsampling procedure involving two independent subsets of data: characterized with high and low statistics. The former one was randomly divided into folds of the same size as the undivided (small) subset. Our variability study was performed on each fold and the mean values of p_a_, p_b_ and p_ab_ along with their standard errors of means (SEM) were computed over all folds. Differences between results obtained from the whole subset and the mean values, as well as the magnitude of SEM allow us to conclude about potential impact of the sampling bias and about estimation errors.

## Results

In this section, we present results of the second-order probability analysis. With this term, we refer to distributions of p_aa_, p_bb_ and p_ab_, whereas with “intrinsic variability” or simply “variability” we will refer to p_ab_ only. In what follows, we particularly focus on how the second-order quantities are affected by the choice of experimental parameters, namely by: (1) the Z-score threshold chosen for the SPES-Rs analysis, (2) stimulating current intensity, (3) distance between stimulating and recording electrode contacts. Strictly speaking, both a stimulation and a recording are performed by two adjacent contacts (see Materials and Methods). By a *stimulating (recording) site* we will mean an averaged position (point in between) of the two stimulating (recording) electrode contacts. Then the distance is measured between the stimulating and the recording sites. We present two sets of results: where a response is considered significant if it crosses the Z-score threshold wherever in the 800 ms time window after stimulation, and where its maximum has to appear within the first 200 ms after stimulation. To the latter condition, we will refer to as “peak delay cut”.

**Fig. 1:**
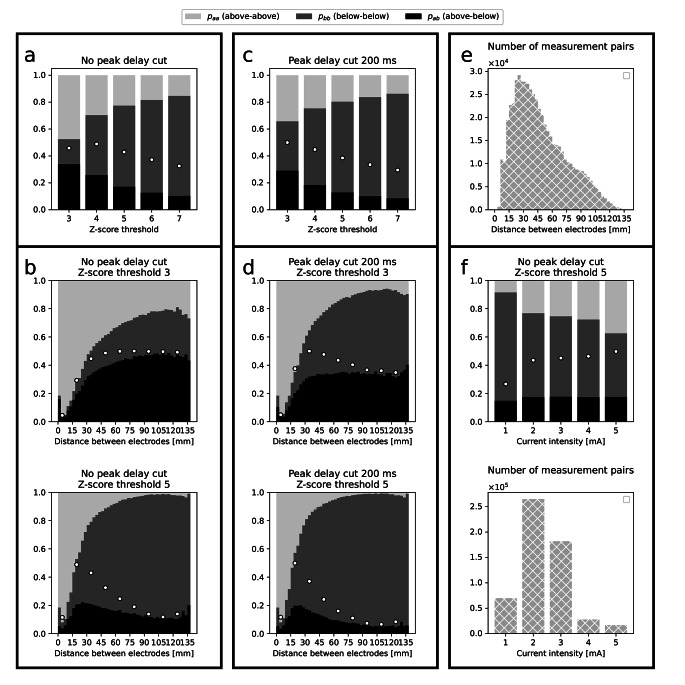
* Second-order probability analysis of paired thresholded responses to two identical SPES. Light gray bars indicate the proportion in all measurement pairs of pairs where both responses were above the Z-score threshold (p*_*aa*_*), dark gray indicates the proportion of pairs where both responses were below that threshold (p*_*bb*_*) and black indicates the proportion of measurement pairs where one response was above and the other one was below that threshold (p*_*ab*_*). The latter value is referred to in text as “intrinsic variability”. White dots mark value p*_*ab*_^*max*^, *i.e. the maximum value of intrinsic variability that it would take if all paired responses were trespassing the threshold in an independent fashion (indicating no correlation between thresholded results in a pair). Frame a shows dependency of p*_*aa*_, *p*_*bb*_*and p*_*ab*_*on the Z-score threshold. Frame b demonstrates dependency of these values on the distance between stimulating and recording sites for Z-score threshold Z=3 (top panel) and Z=5 (bottom panel). Frames c and d show the same as a and b respectively, but with peak delay cut equal to 200 ms. Panel e shows statistics relevant to frames b and d. In the case of these frames for the sake of clarity, p*_*ab*_^*max*^*is plotted only for every fifth bin (white dots). Frame f shows dependence of p*_*aa*_, *p*_*bb*_*and p*_*ab*_*on stimulating current intensity (top panel) and the relevant statistics (bottom panel)*

First, we studied the importance of the choice of the Z-score threshold in the second-order probability analysis. Figure 1a shows that raising the threshold entails an increase of the relative number of bb measurement pairs (dark gray bar) and a decrease of the number of the aa pairs (light gray). Intrinsic variability is relatively closest to its maximal value (white dots) for the lowest Z-score threshold and it decreases with the growing threshold.

Second, we investigated the effect of the Euclidian distance between stimulating and recording sites. This effect is presented in Fig. 1b top and bottom panels for Z-score thresholds Z = 3 and Z = 5 respectively. The first panel shows that intrinsic variability grows with distance and saturates above ~ 75 mm at the level ~ 0.45 which almost reaches its maximal value (white dots). To the contrary, for Z = 5 (Fig. 1b, bottom) intrinsic variability initially grows with distance and above 25 mm it slightly decays, to finally flatten and almost reach the value of p_ab_^max^ (white dots) around 90 mm. The two above discussed points of the analysis are repeated with peak delay cut equal to 200 ms. Z-score threshold dependence is shown in Fig. 1c and distance dependence in Fig. 1d. These figures show that the peak delay cut reduces intrinsic variability that still roughly reaches its maximal value for large distances. In all distance studies, the first bin is an outlier characterized with low statistics as indicated in Fig. 1e that shows the number of pairs considered per distance in panels b and d. As indicated in this panel, also the largest distances are characterized with relatively low statistics.

Third, we focused on the dependence of the second-order quantities on the stimulating current intensity. This dependence, studied for Z-score threshold Z = 5, is shown in the top panel of Fig. 1 f. This panel demonstrates that variability does not depend on intensity, but p_aa_ and p_bb_ do: as could be intuitively anticipated, higher intensity yields more significant responses. This, in turn, entails an increase of maximal variability (white dots). Next, we applied the subsampling procedure described in Materials and Methods. Stimulations performed with current intensity 2 mA (5 mA) were used as the large (small) subset. The differences between results computed on all data and means computed on folds, as well as standard errors of means, were only non-zero on the third or fourth decimal place. We therefore omit error bars in plots.

**Fig. 2:**
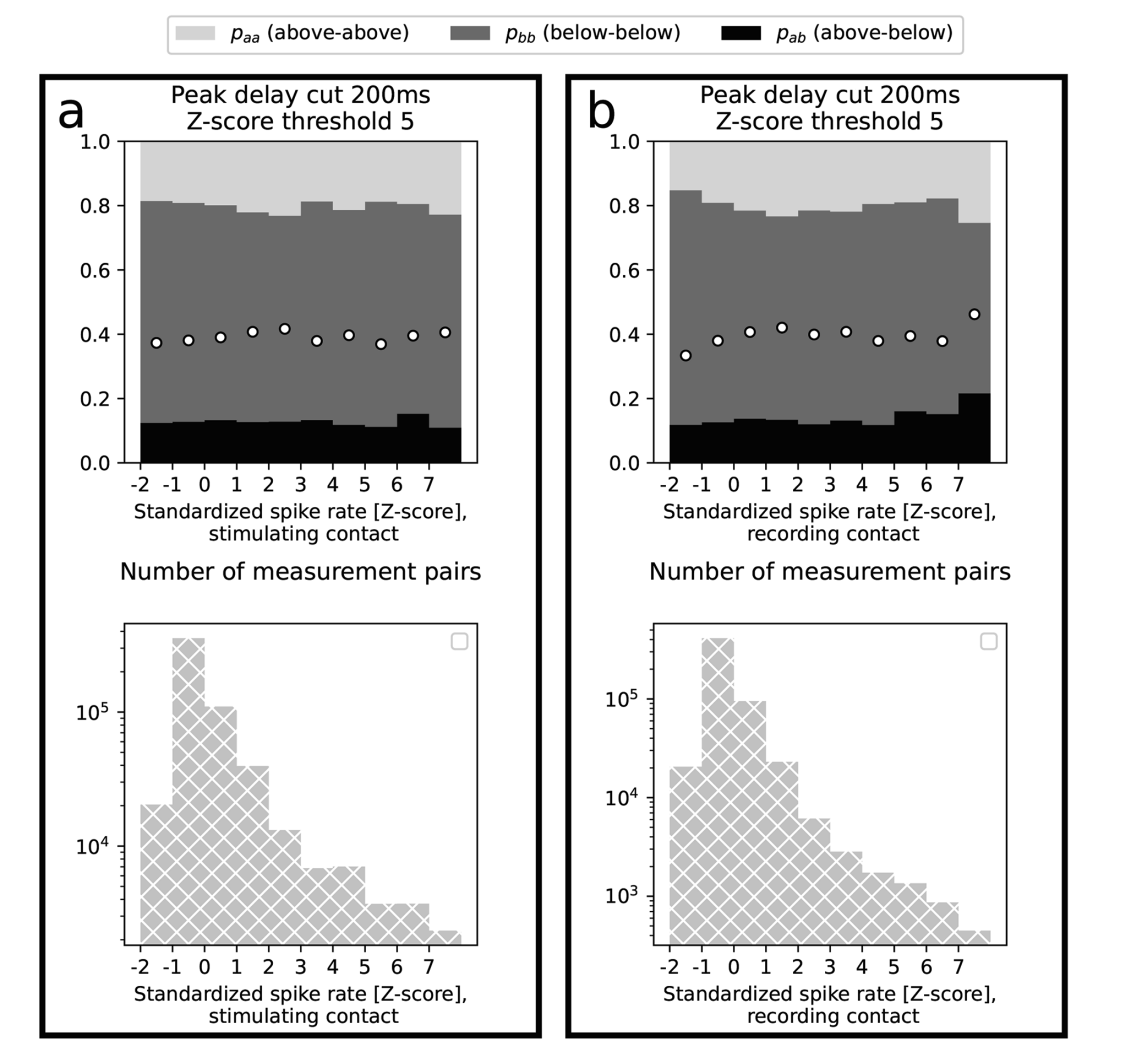
*Dependence of variability on standardized spiking rates of the stimulating (panel a) and the recording (panel b) contacts. Light gray bars indicate the proportion in all measurement pairs of pairs where both responses were above the threshold Z=5 (p*_*aa*_*), dark gray indicates the proportion of pairs where both responses were below that threshold (p*_*bb*_*) and black indicates the proportion of measurement pairs where one response was above and the other one was below that threshold (p*_*ab*_*). White dots mark value p*_*ab*_^*max*^, *i.e. the maximum value of intrinsic variability that it would take if all paired responses were trespassing the threshold in an independent fashion. The bottom panels show statistics relevant to the dependence presented in the top panels*

Fourth, we confirmed that the Z-score threshold and distance studies (for Z = 5), performed five times for all current intensities independently preserve the same trends. Therefore, we did not include multivariate analysis in this study, although bivariate distributions are not strictly factorisable. We note, however, that for short distances (< 20 mm) it is mainly the intensity 1 mA that contributes to occurrence of the ab and bb pairs and that the curve separating p_aa_ and p_ab_ distributions in the distance study becomes less concave with growing intensity (results not shown). Finally, we found that variability is not dependent on spiking rates, as detected by DELPHOS, characterizing either the stimulating (top panel in Fig. 2a) or the recording electrode contacts (top panel in Fig. 2b).

## Discussion

In this paper, we presented second-order probability analysis of responses to single pulse electrical stimulations (SPES-Rs) performed and recorded by SEEG electrodes implanted in drug-resistant epileptic patients and candidates to brain resection surgery. Thereby we studied variability of thresholded responses performed twice between the same electrode contacts and in the same conditions. Our results inform about variability and thus repeatability of brain functional connections obtained from stimulation-based SEEG measurements, such as those leading to the derivation of a whole-brain functional connectivity atlas (Trebaul et al., 2018).

This atlas is *probabilistic* in the frequentist sense, because it averages binarized significance of corresponding responses recorded in many patients. It was computed on the F-TRACT dataset, the same as we used for this study. As of today, this dataset aggregates data from 29 medical centers that use different clinical protocols. In effect, the averaged results comprise measurements obtained in different conditions, between which, for example, durations of the stimulating runs could differ. Furthermore, the data were collected in clinical conditions less strictly controlled than e.g. in the case of cognitive research and therefore we can not report the exact state of patients, who were nevertheless asked to lay still. Finally, individual differences between subjects diversify the results. For these reasons a certain degree of variability could have been anticipated. Up to now, however, variability of measurements performed on the same subject, in the same conditions and under the same medical treatment was not known. Although in our study we did not control or consider all the conditions of the data recorded in clinical setting or associated constraints, our results shed light on the intra-subject variability and showed how and to what extent it could be controlled.

In particular, we defined and quantified intrinsic variability p_ab_ that informs about changeability between two thresholded measurements performed in the same patient and in the same conditions. When this quantity reaches its maximum value p_ab_^max^ that we found theoretically, it is a signature of independence between the two measurements. Then, each measurement will be significant with the same probability p_a_ regardless of the result of the other measurement. Because the first and the second measurements are, on average, characterized with the same p_a_ (see Materials and Methods), intrinsic variability along with another second-order quantity, e.g. p_aa_, allows to compute other values of interest, for example probability that one measurement will be significant if such was the other one. It is given by conditional probability: p_aa_ / p_a_ = p_aa_ / (p_aa_ + 0.5 · p_ab_). An analogous formula can be derived for insignificance. Similarly, Pearson correlation between measurements can be found as 1 - p_ab_ / p_ab_^max^.

We studied how p_aa_, p_bb_ and p_ab_ change with chosen factors. First, we focused on the effect of the choice of the Z-score threshold. Results presented in Fig. 1a lead us to conclude that Z = 3 is too low, as in this condition intrinsic variability reaches 0.35 being not far from its maximal value (0.45). For Z = 5 correlation grows above 0.5, which could be a criterion for the threshold choice. One could also use thresholds greater than 5, but, as indicated in Fig. 1a, this comes at the cost of lowering the number of detected significant responses. Therefore, we conclude that Z = 5 might be a reasonable choice. Introducing an additional constraint that only responses arriving in a 200 ms window after stimulation are significant, leads to correlation trespassing 0.5 already for Z = 4 (Fig. 1c).

Second, we showed that for long distances between stimulating and recording electrode contacts ( > ~ 90 mm), the intrinsic variability almost reaches its maximal value, which we interpret in what follows. The relative difference between p_ab_^max^ and p_ab_ equals the value of the Pearson correlation. In the case of Z = 3 without the peak delay cut (Fig. 1b top) this correlation for all pairs separated by at least 90 mm equals 0.04. We confirmed this result by computing correlation explicitly on two measurement series and we found the associated p-value to be negligible. Note that the magnitude of this correlation informs about deviation from independence, not about similarity of paired measurements that can be high, even when Pearson correlation is small. This similarity for long distances and conditions presented in the top panel of Fig. 1b, is reflected in the fact that the probability of repeating an (in)significant measurement is (0.48) 0.56. Lifting the threshold up to Z = 5 and introducing the peak delay cut (Fig. 1d bottom) for long distances leads to a decrease of p_ab_ and p_ab_^max^, but at the same time to an increase of their relative difference – thus correlation – to 0.25. In these conditions, the probability of repeating an (in)significant measurement is (0.97) 0.28. Near to zero correlation for long distances and Z = 3 corroborates our earlier conclusion that this value of the threshold is too low.

The decay of p_aa_ with distance, shown in Fig. 1b and d, is consistent with the decay of response amplitude observed in other studies (Trebaul et al., 2018; Silverstein et al., 2020). We hypothesize that one reason for the decay of p_aa_ could be a polysynaptic character of long-range connections. If any monosynaptic connection is realized with some probability, then the probability of completing the whole polysynaptic route drops with the number of links. On the other hand, connections performed over the whole network could be realized over various paths. These two effects together combined with network reconfiguration between the measurements could be the reason for the decay of correlation on long distances. Contribution to p_aa_ on long distances could be due to interhemispheric homotopic connections, which were shown to be relatively strong (Trebaul et al., 2018). To sum up, this part of our results uncovers variability of long-range functional connections that could be intrinsic to the human brain but also could follow from limitations of the SPES-R method.

In the last part of our study we found that higher stimulating current intensity leads to an increase of p_aa_ (Fig. 1f). It can be intuitively anticipated, as it is known that higher stimulation current leads to greater amplitude of response (Donos et al., 2016; Trebaul et al., 2018; Hays et al., 2021). Nevertheless, our results provide more insight than that. Figure 1f shows that intrinsic variability does not depend on the stimulation current, which could be interpreted in the following way: if, due to an increase of intensity, p_ab_ decreases in favor of p_aa_, then this effect is compensated by the decrease of p_bb_ in favor of p_ab_. Nevertheless, variability changes only slightly between intensities 2 and 4 mA. Intensity 1 mA might be too low, since it yields correlation lower than 0.5. Then, we used the intensity study to engage a subsampling procedure allowing us to verify that the sampling bias does not affect the results. We also found estimation errors to be negligible. Finally, we did not observe any dependence of our results on the interictal spike rates of stimulating or recording contacts. Although the studies about the link between this rate and the location of the epileptic zone are still ongoing (Bartolomei et al., 2016), this result suggests that our findings are robust with respect to epileptic pathologies.

In summary, in this paper we presented a second-order probability analysis, in which we found that the probability of observing two discrepant SPES-Rs, obtained with a widely accepted data analysis protocol, can be substantial. We characterized conditions conducive for this effect and we conclude that data should be interpreted in a probabilistic manner even in the context of a single individual. Here we showed that interpretations assuming permanency of responses significance drawn from individual SPES-R readouts are likely to be inaccurate in certain conditions. Our results allow for more informed interpretation of SEEG-derived functional connectivity and improved experimental setup design. In this study, we were comparing paired measurements, thus responses to stimulations performed twice in the same technical conditions. In the future, more than two identical stimulations could be examined, which could allow for derivation of response amplitude distributions and probabilities already on the individual level. Moreover, future analyses could include the timing of stimulations and the number of delivered single pulses. Studies focused on changes to brain states could be concerned with correlating variability of SPES-Rs with factors such as subject’s condition or performance in a task. Finally, it appears very relevant to carefully control the states of the patients when they are stimulated, to minimize the variability in SPES-Rs of biological origin.
